# Higher CO_2_ Assimilation in Selected Rice Recombinant Inbred Lines Is Driven by Higher CO_2_ Diffusion and Light Use Efficiency Related to Leaf Anatomy and Mesophyll Cell Density

**DOI:** 10.3389/fpls.2022.915050

**Published:** 2022-06-09

**Authors:** Faliang Zeng, Lin Zhu, Guojiao Wang, Yinpei Liang, Dianrong Ma, Jiayu Wang

**Affiliations:** Key Laboratory of Rice Biology & Genetic Breeding in Northeast China (Ministry of Agriculture and Rural Areas), Rice Research Institute, Shenyang Agricultural University, Shenyang, China

**Keywords:** leaf anatomy, CO_2_ diffusion, stomatal conductance, mesophyll conductance, electron transport rate, mesophyll cell number, photosynthesis

## Abstract

Leaf anatomy determining the light distribution within the leaf and exerting influence on CO_2_ diffusion is considered to have dramatic potential for photosynthesis performance increase. In this study, we observed that two rice recombinant inbred lines, H138 and H217 (RILF_11_ plants from Sasanishiki × IRAT10), have higher net CO_2_ assimilation (An) than their parent Sasanishiki due mainly to the improvement of leaf anatomy. Our results showed that An positively correlated with anatomy traits’ mesophyll cell number per cross-sectional area (NO_.mescell_/A_cros_) and mesophyll area (A_mes_). NO._mescell_/A_cros_ exert direct and indirect effects on An. Compared to Sasanishiki flag leaves, IRAT10, H138, and H217 have higher mesophyll cell numbers. Simultaneously, higher chlorophyll content and expression of genes encoding the light-harvesting protein of PSII and PSI (*Lhcb1*, *2*, *3* and *Lhca1*, *2*, *3*) were recorded in IRAT10, H138, and H217, which facilitates light use efficiency. Higher electron transport rate and RuBP concentration were recorded in IRAT10, H138, and H217 flag leaves. Retinoblastoma-related gene (*OsRBR1*), exerting effects on mesophyll cell density, can be used to modify leaf anatomy for improving leaf photosynthesis. Additionally, higher stomatal conductance and mesophyll conductance were also recorded in H138 and H217 than in Sasanishiki. Furthermore, we modeled mesophyll conductance through anatomical traits, and the results revealed that chloroplast thickness was the dominant factor restricting CO_2_ diffusion within mesophyll cells rather than cell wall thickness. Higher RuBP content accompanied by higher CO_2_ concentration within the carboxylation set in H138 and H217 flag leaves contributed to higher CO_2_ assimilation.

## Introduction

Rice is an important food crop, and its yield mainly derives from the accumulation of photosynthetic substances in leaves after the heading stage. Photosynthesis, a key factor in the formation of rice yields, contributed little to rice productivity gains over the past half-century (Zhu et al., 2010). Improving the photosynthetic efficiency of crop leaves has been considered an important and effective strategy for increasing crop yield potential and productivity (Zhu et al., 2010; Lawson et al., 2012; Ort et al., 2015; Simkin et al., 2019). Leaf anatomy determining the light distribution within the leaf and exerting dramatic influence on CO_2_ diffusion is considered to have dramatic potential on the enhance of photosynthetic capacity (Zhang et al., 2014; He et al., 2017; Lehmeier et al., 2017). Thicker leaves with more and small mesophyll cells could increase mesophyll conductance (g_m_), which contributes to a higher photosynthesis rate (He et al., 2017). Meanwhile, wild rice with fewer larger mesophyll cells between two minor veins, but with more chloroplasts in a mesophyll cell, contributed to higher photosynthetic efficiency (Mathan et al., 2021). Both larger and smaller mesophyll cells increase the net photosynthetic rate in Arabidopsis, while the mechanism of photosynthesis enhancement differs in different mesophyll cell sizes (Barroco et al., 2006; Lehmeier et al., 2017). Additionally, rice with *Narrow leaf1* (*NAL1*) genes changed the arrangement of vascular bundles in leaves and narrowed the leaves, which achieved higher photosynthesis efficiency (Zhang et al., 2014). Hence, the precise target for engineering leaf anatomy still needs to be further studied.

Accordingly, CO_2_ diffusion within the leaf can be divided into stomatal diffusion and mesophyll diffusion. Stomatal diffusion mainly refers to the diffusion of CO_2_ moving from the atmosphere to the sub-stomatal internal cavities through the stomatal pores. Mesophyll diffusion refers to the diffusion of CO_2_ moving from sub-stomatal internal cavities to the outside the mesophyll cell wall (gas phase diffusion), and from there to the site of carboxylation inside the stroma through the cell wall, plasma membrane, cytoplasm, chloroplast envelope membranes and stroma (liquid phase diffusion) (Evans et al., 1994, 2009). Mesophyll conductance has been recognized as a crucial limitation, and even the most consistent limitation of photosynthesis in some species (Tosens et al., 2012a,b; Peguero-Pina et al., 2017; Xiong et al., 2017; Xiong and Flexas, 2018; Han et al., 2018; Ren et al., 2019; Flexas et al., 2021). However, g_m_ varied across species, even the same species between genotypes mainly linked to anatomical properties (Tomas et al., 2013; He et al., 2017; Ouyang et al., 2017; Peguero-Pina et al., 2017). Among leaf anatomical traits, mesophyll cell wall thickness (T_cw_) and the total chloroplast surface area exposed to mesophyll intercellular air spaces per leaf area (S_C_/S) are the two strongest anatomical limitations to the maximum gm (Tomas et al., 2013; Evans, 2021). The cell wall thickness strongly and negatively correlates with gm and An, and limitations derived from S_C_/S are frequently equal to or smaller than T_cw_ (Flexas et al., 2021). High S_C_/S means more chloroplast exposed to CO_2_, which can improve CO_2_ concentration at the sites of carboxylation by a great level (He et al., 2017). However, a recent study showed that T_cw_ and Sc/S seemed to be varied independently, and the two factors compensate for each other to achieve the maximum gm in some cases (Peguero-Pina et al., 2017). Moreover, in cotton leaves, T_cw_ is the dominant factor restricting gm in palisade tissue cells, while chloroplast thickness (T_chl_) is the main liquid component restricting gm in the sponge tissue (Han et al., 2018). Furthermore, the drought did increase T_cw_ of rice but was not followed by a decrease in gm, which might indicate that T_cw_ was not the dominant limiting factor for gm under water stress (Ouyang et al., 2017). This view has not been proven yet. Hence, the dominant liquid factor restricting CO_2_ diffusion in rice still needs to be researched.

Under the field condition, we found that IRAT10 with thicker leaves has a higher photosynthetic rate than Sasanishiki, which may be benefited from leaf anatomy traits. Additionally, H138 and H217 from the recombinant inbred line of IRAT10 × Sasanishiki have higher photosynthetic rates than their parent Sasanishiki. We suspect that the increase in photosynthetic efficiency of H138 and H217 might be related to the leaf anatomical traits inherited from their parent IRAT10. This has great significance for rice breeding. It makes sense to investigate the mechanism of photosynthetic improvements benefited from CO_2_ diffusion or biochemical factors mediated by leaf anatomy traits. In addition, we modeled mesophyll conductance from anatomical traits to reveal the dominant components that restrict CO_2_ diffusion and make up for the lack of application of the methods in rice.

## Materials and Methods

### Plant Material and Grown Condition

Sasanishiki (*Oryza sativa japonica* subspecies) and IRAT10 (*O. sativa japonica* subspecies, upland), as well as two recombinant inbred lines, H138 and H217 (RILF11 plants from Sasanishiki × IRAT10), were used in this study. The genetic map of tested materials is shown in Supplementary Figure 1. Seeds were pre-germinated and grown in a nursery for four weeks, and then the seedlings were transplanted into 11-L plots, containing 10-kg paddy soil, with a density of two plants per hill and two hills per pot. Fertilizers, weeds, pests, and diseases were controlled the same with the field to avoid a yield loss. For each genotype, 40 pots were planted. At the heading stage (14 days after flag leaves full expansion, on August 3, 2020), the fully expanded flag leaves on the main stem were sampled and measured on the same day to identify the difference between the two cultivars on photosynthesis performance. The heading stage of the tested varieties was almost the same.

### Chlorophyll Content, Gas Exchange, and Chlorophyll Fluorescence

Chlorophyll was extracted from flag leaves in 20 ml of 95% alcohol for 48 h at room temperature under a dark environment according to the method described by Sartory and Grobbelaar (1984). The absorbances of the extract under 665 and 649 nm were determined using a spectrophotometer. Chlorophyll content were calculated with the following equations: chlorophyll a (μg mL^–1^) = 13.95 (A_665_) – 6.88 (A_649_), chlorophyll b (μg mL^–1^) = 24.96 (A_649_) – 7.32 (A_665_). The total chlorophyll content is the sum content of chlorophyll *a* and chlorophyll *b*. For each genotype, six biological replicates were measured.

A portable infrared gas analyzer (CIRAS3, PP Systems, Amesbury, MA, United States) equipped with an integrated leaf fluorometer head (CFM3, PP Systems) was used to measure gas exchange and chlorophyll fluorescence parameters simultaneously on a sunny day. About nine flag leaves on the main stem of each genotype were selected to exert gas exchange measurement. The photon flux density (PPFD) in the leaf chamber was maintained at 1,200 μmol m^–2^ s^–1^ provided by a LED source with a light ratio of red: green: blue: white 90: 0: 5: 5%. CO_2_ concentration in the leaf chamber was maintained at 400 μmol mol^–1^ provided by a CO_2_ injector system. The leaf temperature in the chamber was controlled at 25°C, and the flow rate was controlled at 200 μmol s^–1^. Net CO_2_ assimilation (An, μmol m^–2^ s^–1^), internal CO_2_ concentration (Ci, μmol mol^–1^), and stomatal conductance (g_S_, mmol m^–2^ s^–1^) were recorded simultaneously when the system stabilized to a steady-state. Then, a fluorometric procedure was performed to measure the steady-state fluorescence (F_S_) and maximum fluorescence (F_m_′). The saturating light pulse was 8,000 μmol m^–2^ s^–1^. The actual photochemical efficiency of photosystem II (ΦPSII) was calculated with the following equations:


(1) $$\Phi PSII=\frac{F_{M}^{\prime }-F_{S}}{F_{M}^{\prime }}$$

The electron transport rate (J) was calculated as:


(2) J=Φ⁢P⁢S⁢I⁢I×P⁢P⁢F⁢D×α×β

where α refers to the leaf absorbance, and β is the distribution of electrons between the photosynthetic system I (PSI) and PSII. Previously, α was assumed as 0.85, and β was assumed as 0.5 between PSI and PSII (Manter and Kerrigan, 2004; Li et al., 2009; Gilbert et al., 2012). In this paper, α and β were estimated from a light response curve under a gradient of PPFD of 400, 200, 100, and 0 μmol m^–2^ s^–1^ at low O_2_ concentrations (<2%). The slope of the relationship between ΦPSII and 4ΦCO_2_ (the quantum efficiency of CO_2_ uptake) was taken as the value of α⋅β (Valentini et al., 1995).

The mesophyll conductance of CO_2_ (g_m_) was determined as the following equation described by Harley et al. (1992):


(3) $$g_{m}=\frac{A}{C\,i\times \frac{\Gamma^{*}\times \left(J+8\,\left(A+R_{d}\right)\right)}{J-4\,\left(A+R_{d}\right)}}$$

where Γ* refers to the CO_2_ compensation point in the absence of photorespiration, and R_d_ is the respiration in the daytime. Γ* and *R*_*d*_ were estimated from A/Ci curves, and CO_2_ concentrations in the leaf chamber were set in a series of 200, 150, 100, 75, and 50 μmol mol^–1^, under three light intensities (300, 200, and 100 μmol m^–2^ s^–1^) and low O_2_ concentrations (<2%) (Li et al., 2009) (Pons et al., 2009). The coordinate of the intersection of the three A/Ci curves represented *R*_*d*_ and Γ*(Li et al., 2009; Pons et al., 2009). CO_2_ leakage was measured to make the gas exchange measurement more accurate (Flexas et al., 2007).

### Analysis of Leaf Morphological and Anatomical Characters

Nine flag leaves on the main stem of each genotype were graphed to measure leaf length, leaf width, and leaf area using Image J software (National Institutes of Health, Bethesda, MD, United States). Dry weights were determined when the flag leaves were dried to a constant weight at 80°C for 48 h. Leaf mass per area (LMA) is the ratio of dry weight and leaf area. Then the dried samples were used for nitrogen concentration determination through a carbon and nitrogen analyzer (MT700 Mark II; Yanako).

For light microscopy, leaf sections (approximately 1 cm in length) from the middle of leaves were collected with the formalin acetic-alcohol solution. Samples were dehydrated with ethanol series (50, 70, 80, 95, and 100%) and then, were embedded with paraffin (melting point 56–58 degrees). A sliding microtome (Leica RM2255) was used to cut the block into transverse sections 7 μm in thickness. Light sections were stained using 1% toluidine blue, then the sections were scanned under a digital whole slide scanning. Anatomical characteristics were determined using CaseViewer.

The thickness of leaf/mesophyll (T_leaf_/T_mes_, μm), the cross-sectional area of each mesophyll cell (A_cell_, μm^2^), the ratio of mesophyll cell (A_mes_), bulfom cell (A_bf_), main vein (A_main vein_), major vein (A_maj vein_), and minor vein (A_min vein_) area to cross-sectional area (A_cros_), and the lengths between two neighboring major/minor vascular bundle (L_major vein_/L_minor vein_, μm), were determined. Mesophyll cell number per cross-sectional area (NO_mes cell_/A_cros_, number μm^–2^) was calculated as a quotient of dividing A_mes_ by A_cell_. The cross-sectional areas of >30 mesophyll cells were measured between the vascular bundles. For each genotype, at least six leaf sections from flag leaves of different main stems were used for anatomical character measurement.

### Electron Microscope and Estimate Gm From Anatomical Characteristics

Leaf pieces between the first and the second major vein (approximately 4 mm × 1 mm) from the same leaves used for light microscopy were cut and immersed in 0.1 M phosphate buffer (pH 7.2) with 2.5% glutaric aldehyde and 3% paraformaldehyde under vacuum for 48 h. Then, the leaf pieces were further fixed in 1% osmium tetroxide for another 2 h, followed by washing three times with phosphate buffer. Then, an ethanol series was used to dehydrate leaf pieces. After dehydration, the leaf samples were embedded in resin for ultrathin sections. Ultrathin sections were further stained with 7.7% uranyl acetate (30 min) and lead citrate (4 min) for photographing under an electron microscope (TEM HT770, Hitachi, Japan). Chloroplast length (L_chl_), chloroplast thickness (T_chl_), and the cross-sectional area of chloroplast (A_chl_) were measured at 1,200–1,500× magnification. Mesophyll cell wall thickness (T_cw_) and cytoplasm thickness (T_cyt_) were measured at 12,000× magnification. For each genotype, six-leaf sections from different plants were used. For each section, three different view fields were measured.

The fraction of intercellular airspace (*f*_*ias*_) was determined as (Tomas et al., 2013):


(4) $$f_{i\,a\,s}=1-\frac{\sum {\text{S}}_{\text{s}}}{{\text{T}}_{\text{mes}}\times \text{W}}$$

where ∑S_s_ is the total area of mesophyll cells in the section, T_mes_ refers to the mesophyll thickness between epidermal layers, W is the section width, and all measurements were performed at 800× magnification.

The surface area of mesophyll and chloroplasts exposed to the intercellular airspaces (S_m_/S and S_C_/S) were calculated as follows (Evans et al., 1994; Scafaro et al., 2011):


(5) $$S_{m}=\frac{L_{m}}{W}\,F$$

(6) $$S_{c}=\frac{L_{c}}{W}\,F$$

The L_m_ and L_c_ refer to the length of mesophyll and chloroplast exposed to the intercellular airspace (μm). F is the curvature correction factor and was taken as 1.55 in rice (Syvertsen et al., 2010).

A one-dimensional gas diffusion model was used to estimate mesophyll conductance from anatomical characteristics (g_m ana_) (Niinemets, 2003; Tosens et al., 2012a,b). In this model, mesophyll conductance is divided into gas-phase conductance and liquid-phase conductance.


(7) $$g_{m\,a\,n\,a}=\frac{1}{\frac{1}{g_{i\,a\,s}}+\frac{R\,T_{k}}{H\cdot g_{l\,i\,q}}}$$

where *g*_ias_ is the gas phase conductance that links to CO_2_ moving from substomatal internal cavities to the outer surface of the mesophyll cell wall, and g_liq_ is the liquid phase conductance that links to CO_2_ moving from the outer surface of cell walls to the stroma. Gas constant (R, Pa m^3^ K^–1^ mol^–1^), Henry’s law constant (H, Pa m^3^ mol^–1^), and absolute temperature (T_k_, K) were used to convert g_liq_ to gas equivalent conductance (Niinemets, 2003).

The gas-phase conductance (g_*ias*_) is determined as (Niinemets, 2003):


(8) $$g_{i\,a\,s}=\frac{D_{a}\times f_{i\,a\,s}}{△\,L_{i\,a\,s}\times \varsigma }$$

where ς, the diffusion path tortuosity, was assumed as 1.57 (mm^–1^); and D_*a*_, the diffusion coefficiency for CO_2_ in the gas phase, was assumed as 1.51 × 10^–5^ (m^2^ s^–1^ at 25°C); ΔL_*ias*_, the average gas-phase thickness, was assumed as half of the mesophyll thickness between epidermal layers.

The total liquid phase conductance is the sum conductance of each component including cell wall (*g*_cw_), plasmalemma (*g*_pl_), cytosol (*g*_cyt_), chloroplast envelope (*g*_en_), and chloroplast stroma (*g*_st_).


(9) $$\frac{1}{g_{l\,i\,q}}=\left(\frac{1}{g_{c\,w}}+\frac{1}{g_{p\,l}}+\frac{1}{g_{c\,y\,t}}+\frac{1}{g_{e\,n}}+\frac{1}{g_{s\,t}}\right)\times \frac{S_{c}}{S}$$

The conductance of the mesophyll cell wall, cytosol, and chloroplast can be estimated from a general equation (Tosens et al., 2012a,b).


(10) $$t\,h\,e\,g_{i}=\frac{r_{f,i}\times D_{w}\times p_{i}}{\Delta \,L_{i}}$$

where dimensionless factor *r*_f,i_ is the reduction of diffusion conductance in corresponding components relative to free diffusion in water. The *r*_*f,i*_ approximate to 1 for cell wall, and 0.3 for g_*cyt*_ and g_*st*_. D_*w*_, the aqueous phase diffusion coefficient for CO_2_, was 1.79 × 10^–9^ (m^2^ s^–1^) at 25°C. ΔL_*i*_ (m) is the corresponding thickness of each component in the CO_2_ diffusion pathways. The effective porosity (pi, m^3^ m^–3^) in cytosol and chloroplast was taken as 1. Cell wall porosity (p_cw_) was calculated from T_cw_ (p_cw_ = -0.3733 × T_cw_+.3378) (Tosens et al., 2016). Both g_*pl*_ and g_*en*_ were assumed as 0.0035 ms^–1^ (Evans et al., 1994; Tosens et al., 2012a,b). Conductance units in the form of m s^–1^ were converted into molar units according to the equation g[mol m^–2^ s^–1^] = g[ms^–1^] × 44.6 × 273.15/(273.15 + Tem_*L*_) × (P/101.325), where Tem_*L*_ is the leaf temperature (°C) and P (Pa) is the air pressure (Tosens et al., 2012a).

### Limitation Analysis of Different Components of g_m_

Limitations of each component involved in CO_2_ diffusion pathways were calculated as (Tosens et al., 2016):


(11) $$L\,m_{i\,a\,s}=\frac{g_{m\,a\,n\,a}}{g_{i\,a\,s}}$$

(12) $$L\,m_{i}=\frac{g\,m_{a\,n\,a}}{g_{i}\times \frac{S_{c}}{S}}$$

where *Lm*_ias_ refers to the limitation derived from the gas phase component, Lm_i_ refers to the limitation of the cell wall, plasmalemma, cytoplasm, chloroplast envelope, and stroma.

### Stomatal Morphological Traits and the Maximum Stomatal Conductance

Another two leaf sections (approximately 5 mm × 5 mm) from the same leaf used for anatomical measurements were collected with the fixative 2.5% glutaric aldehyde in 0.1 mol L^–1^ phosphate buffer (pH 7.6) under dark for 48 h. Then, the leaf samples were washed three times with phosphate buffer. After an ethanol series and a tertButanol series, the samples were freeze-dried. Images were photographed using a scanning electron microscope (HITACHI Regulus 8100). At least three views on abaxial and adaxial sides were photographed from one section, and at least six leaves from different plants were used for each genotype. Stomatal density (SD, NO. mm^2^), stomatal length (L_S_, μm), and stomatal width (W_S_, μm) on each leaf side was measured using ImageJ. The stomatal size was determined by the maximum area of the open stomatal pore (a_*max*_, μm^2^) calculated as described in Ouyang et al. (2017).


(13) $$a_{m\,a\,x}=\frac{\left(L_{S}\times W_{S}\times \pi \right)}{4}$$

Maximum theoretical stomatal diffusive conductance (g_S max_) can be estimated according to,


(14) $$g_{S\,m\,a\,x}=\frac{d\times S\,D\times a_{m\,a\,x}}{1.6\times v\times \left(l+\frac{\pi }{2}\times \sqrt{\frac{a_{m\,a\,x}}{\pi }}\right)}$$

where *d* is the diffusivity of water vapor in the air (24.9 × 10^–6^ m^–2^ s^–1^, at 25°C), *v* is the molar volume of air (22.4 × 10^–3^ m^3^ mol^–1^, at 25°C), and *l* is the stomatal pore depth for fully open stomata, which assumed to be equal to the width of stomatal guard cell. The g_*S max*_ is the sum of abaxial and adaxial conductance.

### Quantitation of Content and Activity of Carbon Fixation Related Enzyme

After the gas exchange measurement flag leaves on the main stem were collected and stored at –80°C until to determine the biochemical indices. For each genotype, six biological replicates were measured. The contents of RuBP, Rubisco, PGA (3-phosphoglycerate), and the activity of Rubisco were measured using commercial ELISA kits following the manufacturer’s instructions (Jiangsu Meimian industrial Co., Ltd.).

### Total RNA Extraction and Real-Time Quantitative PCR Analysis

Flag leaves of tested varieties (4 varieties × 3 biological replicates) were used for total RNA isolation using MiniBEST Plant RNA Extraction Kit (TaKaRa, Dalian, China). Then, total RNA was used for complementary DNA (cDNA) library construction. Real-time PCR was performed on an Applied Biosystems QuantStudio 3 Real-Time PCR System (Thermo Fisher Scientific, United States) using TB Green^®^ Premix Ex Taq™ II (Tli RNaseH Plus) (TaKaRa, Dalian, China). Genes related to photosynthesis were checked, and the sequences of primers are shown in Supplementary Table 1. The relative expression of genes was calculated through method Livak and Schmittgen (2001).

### RNA-Sequencing

Flag leaves of tested varieties (4 varieties × 3 biological replicates) were used for total RNA isolation on the same day after gas exchange measurements. Total RNA was isolated for cDNA library construction. The cDNA libraries were sequenced using Illumina HiSeq™ 2500 sequencing platform (Illumina Crop., San Diego, CA, United States) by MetWare (MetWare Biotechnology Co., Ltd., Wuhan, China). After filtrating the adapter and low-quality reads, clean reads were aligned to the reference genome Nipponbare *O. sativa ssp. japonica*^1^ by DESeq2 (Love et al., 2014; Varet et al., 2016), and the expression abundance was calculated by featureCounts (Liao et al., 2014). Genes with an expression |Log2 Fold Change| ≥ 1 and FDR < .05 were considered as differentially expressed genes (DEGs). Then, DEGs were subjected to enrichment analysis of the clusters of orthologous groups of proteins (KOG data base were used) function and Kyoto Encyclopedia of Genes and Genomes (KEGG) pathway.

### Statistical Aalysis

One-way analysis of variance (ANOVA) was used to test the statistically significant differences among tested varieties, which was performed through SPSS 22.0 software (IBM Inc.). Multivariate correlation analysis was used to reveal the relationship between leaf anatomical traits and photosynthetic parameters. Structural equation modeling (SEM) was performed to determine the causal relationships between leaf anatomy and photosynthetic capacity, which was exerted with AMOS 22.0.0 software.

## Results

### Leaf Anatomical Traits

**FIGURE 1:**
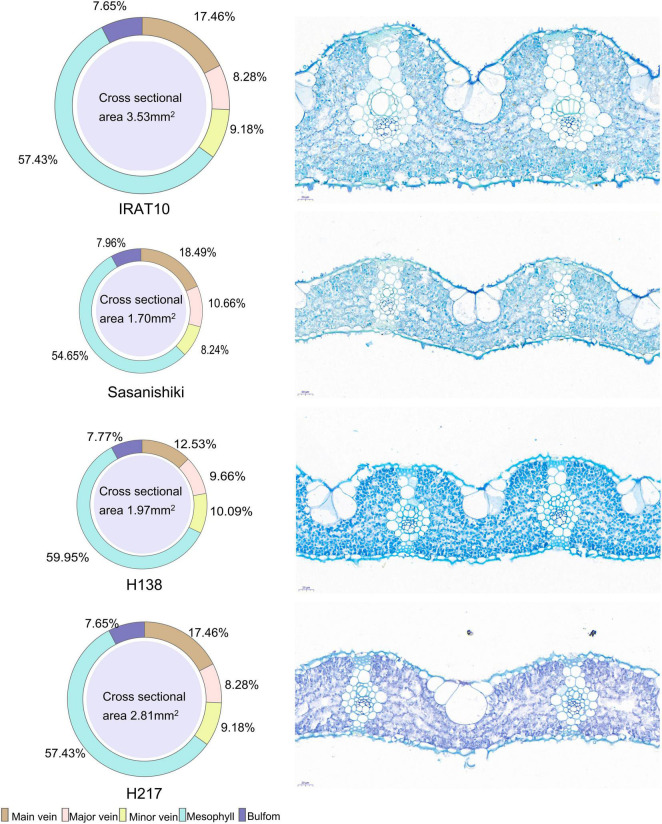
The phenotype of flag leaves of tested varieties. The cross-sectional area of the flag leaf and the proportion of each tissue are shown on the **left**. Slice images are shown on the **right**.

Sasanishiki flag leaves have the longest leaf length (21.08 cm) and H217 has the shortest (29.69 cm) (Table 1). Contrary to leaf length, Sasanishiki flag leaves leaf width was the narrowest (1.22 cm), and H217 was the widest (1.74 cm) among tested varieties. Compared to Sasanishiki, H138, and H217, leaf area and leaf mass per area in IRAT10 flag leaves were the largest (25.96 cm^2^, 6.76 mg cm^–2^). Much longer distances between two neighboring major veins or minor veins (L_major vein_/L_minor vein_) were observed in H217 (1622, 276 μm) flag leaves. The cross-area of a single mesophyll cell varied from 157 to 249 μm^2^, with IRAT10 having the largest and H138 having the smallest. Leaf thickness and the mesophyll thickness of IRAT10 (182, 128 μm), H138 (122, 79.57 μm), and H217 (115, 73.03 μm) flag leaves were significantly thicker than that of Sasanishiki (100, 64.16 μm). The cross-sectional area, the proportion of mesophyll cell area accounts for the cross-sectional area, and mesophyll cell number per cross-sectional area of flag leaves in IRAT10, H138, and H217 were significantly higher than that in Sasanishiki (Figure 1 and Table 1).

**TABLE 1:** Leaf length (L_*l*_, cm), leaf width (L_*w*_, cm), leaf area (A_leaf_, cm^2^), leaf mass per unit area (LMA, mg cm^–2^), the distance between two major veins (L_major vein_, μm), the distance between two minor veins (L_minor vein_, μm), the cross-area of a single mesophyll cell (A_mescell_, μm^2^), the mesophyll cell number per cross-sectional area (NO_.mes_ /A_*cros*_), leaf thickness (T_leaf_, μm), and mesophyll thickness (T_mes_, μm) in the flag leaves of the tested varieties.

	IRAT10	Sasanishiki	H138	H217
Ll (cm)	25.13 ± 2.03*b*	29.69 ± 2.38*a*	23.51 ± 2.58*c*	21.08 ± 2.9*c*
Lw (cm)	1.59 ± 0.06*b*	1.22 ± 0.07*d*	1.44 ± 0.05*c*	1.74 ± 0.11*a*
Aleaf (cm2)	25.961 ± 2.35*a*	23.66 ± 3.31*b*	22.12 ± 2.92*b*	23.75 ± 3.01*b*
LMA (mg cm-2)	6.76 ± 0.16*a*	5.39 ± 0.16*c*	5.88 ± 0.13*b*	5.33 ± 0.07*c*
Ames cell (μm2)	249 ± 13.4*a*	203 ± 7.1*b*	157 ± 6.5*c*	199 ± 4.7*b*
NO.mes cell/Acros	7293 ± 169*b*	4565 ± 161*c*	7537 ± 332*b*	8120 ± 344*a*
Lmajor vein (μm)	1304 ± 12.5*c*	1370 ± 20.5*b*	1294 ± 56.8*c*	1622 ± 75.3*a*
Lminor vein (μm)	217 ± 14.4*b*	213 ± 4.8*b*	184 ± 2.5*c*	276 ± 0.9*a*
Tleaf (μm)	182 ± 5.79*a*	100 ± 0.63*d*	122 ± 2.4*b*	115 ± 1.8*c*
Tmes (μm)	128.91 ± 0.76*a*	64.16 ± 0.57*d*	79.57 ± 2.45*b*	73.03 ± 1.23*c*

*Different letters in the same row indicate significant statistical differences among tested varieties at the 0.05 probability level.*

Chloroplast cross-sectional area (A_chl_) and chloroplast thickness (T_chl_) in Sasanishiki flag leaves (15.15 μm^2^, 3.15 μm) were significantly higher than that of IRAT10, H138, and H217 (Table 2 and Supplementary Figure 2). Chloroplast length (L_chl_) in flag leaves of IRAT10, Sasanishiki, and H138 was 13.8, 10.9, and 12.8% larger than H217 (4.69 μm), respectively. The significantly bigger A_chl_ of Sasanishiki flag leaves was mainly due to the thicker chloroplast thickness (T_chl_), and shorter L_chl_ contributed to a smaller A_chl_ in H217 flag leaves. The number of chloroplast per mesophyll cell (NO_chl_/Cell) has no difference among tested varieties. Cytoplasm thickness (T_*cyt*_) of IRAT10 (0.161 μm) and H217 (0.178 μm) were significantly higher than Sasanishiki (0.110 μm) and H138 (0.134 μm). Compared to Sasanishiki (0.224 μm) and H217 (0.238 μm), IRAT10 (0.189 μm) and H138 (0.216 μm) have a thinner cell wall thickness (T_cw_). The surface area of mesophyll and chloroplasts exposed to the intercellular airspaces (S_m_/S and S_C_/S) and the fraction of intercellular airspace of Sasanishiki, H138, and H217 were significantly higher than that of IRAT10 (6.08, 4.48 μm^2^ μm^–2^, 6.95%).

**TABLE 2:** The cross-sectional area of chloroplast (A_chl_, μm^2^), the chloroplast thickness (T_chl_, μm), the chloroplast length (L_chl_, μm), the number of chloroplast per mesophyll cell (NO._chl_/Cell), the thickness of the cytoplasm between the cell membrane and the chloroplast (T_cyt_, μm), mesophyll cell wall thickness (T_cw_, μm), the surface of mesophyll cells and chloroplasts exposed to leaf intercellular air spaces (Sm/S and Sc/S, μm^2^μm^–2^), the volume fraction of intercellular air space (*f*_ias_, %), and gm modeled from anatomical traits (g_m *ana*_, mol m^–2^ s^–1^) in the flag leaves of tested varieties.

	IRAT10	Sasanishiki	H138	H217
A_chl_ (μm^2^)	10.04 ± 3.11*bc*	15.15 ± 4.88*a*	11.42 ± 3.70*b*	9.42 ± 3.68*c*
T_chl_ (μm)	2.31 ± 0.44*b*	3.15 ± 0.74*a*	2.55 ± 0.75*b*	2.32 ± 0.68*b*
L_chl_ (μm)	5.34 ± 1.07*a*	5.20 ± 1.29*ab*	5.29 ± 0.97*a*	4.69 ± 0.95*b*
NO._chl_/Cell	8.8 ± 1.12*a*	8.3 ± 1.9*a*	8.6 ± 1.1*a*	8.4 ± 1.0*a*
T_cyt_ (μm)	0.161 ± 0.041*a*	0.110 ± 0.036*b*	0.134 ± 0.052*b*	0.178 ± 0.059*a*
T_cw_ (μm)	0.189 ± 0.035*b*	0.224 ± 0.072*a*	0.216 ± 0.055*ab*	0.238 ± 0.053*a*
Sm/S	6.08 ± 0.82*c*	7.41 ± 1.0*a*	7.28 ± 0.60*a*	6.69 ± 0.11*b*
Sc/S	4.48 ± 0.99*d*	7.13 ± 0.88*a*	6.24 ± 0.78*a*	6.08 ± 0.35*a*
*f*_ias_ (%)	6.95 ± 1.49*c*	11.29 ± 1.81*b*	10.22 ± 1.38*b*	14.84 ± 1.88*a*
gm_ana_ (mol m^–2^ s^–1^)	0.108 ± 0.003*a*	0.081 ± 0.001*c*	0.097 ± 0.003*b*	0.104 ± 0.003*a*

*Different letters in the same row indicate significant statistical differences among tested varieties at the 0.05 probability level.*

### Conductance of CO_2_ Diffusion Through Stomatal and Mesophyll Cell

**FIGURE 2:**
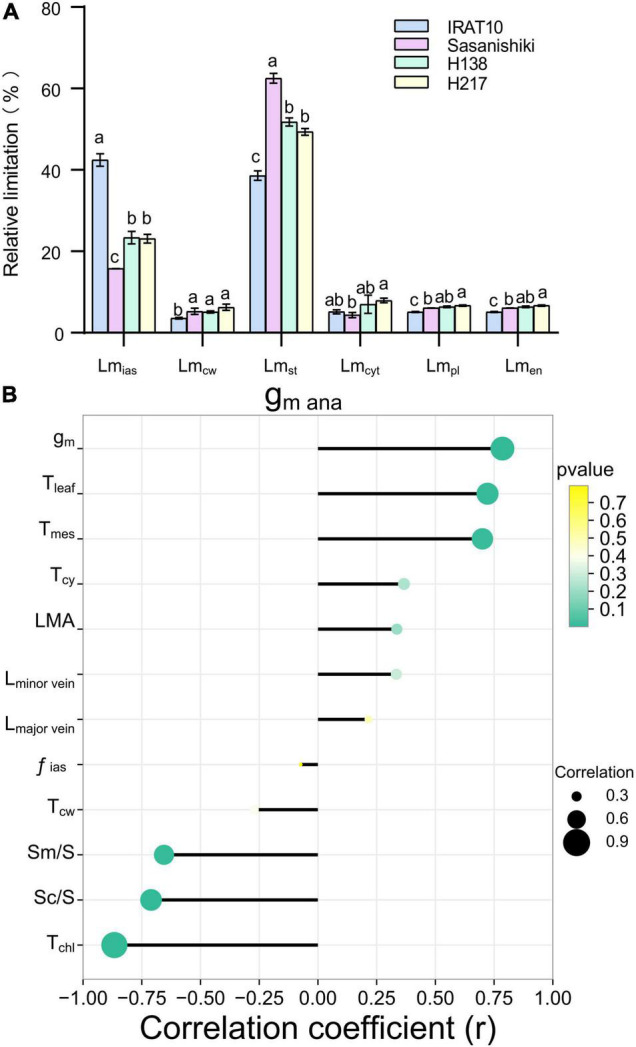
Limitation analysis of modeled mesophyll conductance and the relationship between mesophyll conductance and leaf anatomical traits. Quantitative analysis of each component of modeled mesophyll conductance (g_m *ana*_) in the flag leaves of tested varieties **(A)**. The limitation derived from the gas phase components (Lm_*ias*_), mesophyll cell wall (Lm_cw_), the stroma (Lm_*st*_), the cytoplast (Lm_*cyt*_), the plasmalemma (Lm_*pl*_), and the chloroplast envelope (Lm_*en*_) were quantified according to Tosens et al. (2016). Values are means ± standard error. Different letters indicate significant statistical differences among tested varieties at the 0.05 probability level. Relationship between mesophyll conductance and leaf anatomical traits **(B)**.

Mesophyll conductance (g_m *ana*_), modeled from anatomical traits, was estimated according to the component diffusion conductance of the corresponding diffusion pathway, and the limitation of each component was calculated. The g_m *ana*_ in flag leaves of IRAT10, H138, and H217 was 33.3, 16.9, and 28.4%, respectively, was remarkably higher than that of Sasanishiki (.081 mol m^–2^ s^–1^) (Table 2). The limitation derived from the gas phase components (Lm_*ias*_), ranging from 15.7 to 42.4%, was lower than the total limitation from liquid phase components in all tested varieties (Figure 2A). A significantly higher limitation of gas-phase was recorded in IRAT10 flag leaves (42.4%) when compared with Sasanishiki (15.8%), H138 (23.4%), and H217 (23.1%). Relative to plasmalemma, cytoplast, chloroplast envelope, and cell wall, limitations derived from stroma (Lm_*st*_) were the main limitations among all components in all cultivars in the liquid phase. The Lm_*st*_ in IRAT10 (38.6%) flag leaves were significantly lower than that of Sasanishiki (62.5%), H138 (51.8%), and H217 (49.4%). Despite the value of gm being dramatically higher than g_*mana*_, a significant positive correlation (*r* = 0.786, *p* < 0.01) was observed between g_*mana*_ and gm (Figure 2B). Also, significantly higher positive correlations were observed between g_*mana*_ and T_leaf_ (r = 0.72) and T_mes_ (r = 0.7). However, T_chl_, Sm/S, and Sc/S negative correlate with g_*m ana*_.

**FIGURE 3:**
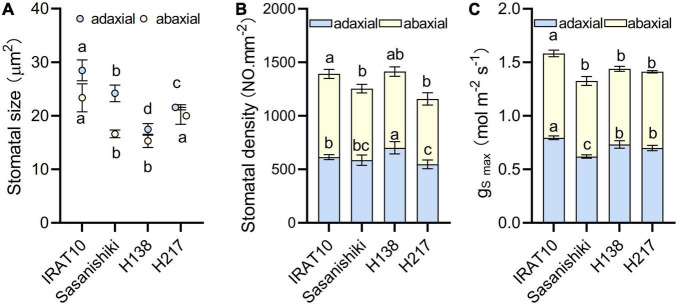
Stomatal size **(A)**, stomatal density **(B)** and the maximum theoretical stomatal diffusive conductance (g_S max_) **(C)** in the flag leaves of tested varieties. Values are means ± standard error. Different letters indicate significant statistical differences among tested varieties at the 0.05 probability level.

Scanning electron microscope results showed that the maximum area of the open stomatal pore (a_*max*_) of IRAT10 flag leaves on the adaxial side and the abaxial side was the largest (28.5, 23.4 μm^2^), and H138 having the smallest (17.5, 15.3 μm^2^) (Figure 3 and Supplementary Figure 3). The variation of stomatal density on the adaxial side of H138 (701 NO. mm^–2^) was significantly higher than that of IRAT10, Sasanishiki, and H217. The variation of stomatal density on the adaxial side ranged from 611 (H217) to 778 NO.mm^–2^ (IRAT10). Maximum theoretical stomatal diffusive conductance (g_S*max*_) was calculated from stomatal density and stomatal area, and the total g_*Smax*_ was the sum of the adaxial and abaxial sides. The total g_*Smax*_ in the flag leaves of IRAT10, H138, and H217 was higher than that of Sasanishiki (1.33 mol m^–2^ s^–1^).

### Photosynthetic Capacity

**FIGURE 4:**
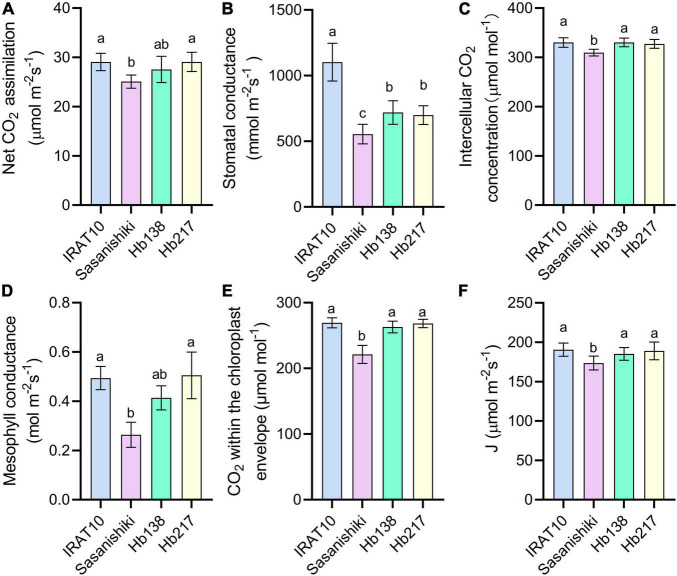
Net CO_2_ assimilation rate (An) **(A)**, stomatal conductance to water (g_S_) **(B)** intercellular CO_2_ concentration (Ci) **(C)** mesophyll conductance (g_m_) **(D)**, CO_2_ concentration within the chloroplast envelope (Cc) **(E)**, and electron transport rate (J) **(F)** in the flag leaves of tested varieties. Values are means ± standard error. Different letters indicate significant statistical differences among tested varieties at the 0.05 probability level.

Relative to Sasanishiki flag leaves (25.1 μmol m^–2^ s^–1^), IRAT10, H138, and H217 have higher CO_2_ assimilation (An) of 29.1, 27.5, and 29.1 μmol CO_2_ m^–2^ s^–1^, respectively, at the heading stage (Figure 4). Stomatal conductance (g_S_), estimated from the gas exchange, in IRAT10, H138, and H217 flag leaves, were 99, 30, and 26%, respectively, significantly higher than Sasanishiki (554 mmol m^–2^ s^–1^). Intercellular CO_2_ concentration (Ci) in flag leaves of IRAT10, H138, and H217 were 7, 6.9, and 5.9%, respectively, significantly higher than Sasanishiki (309 μmol mol^–2^). Mesophyll conductance (g_m_) was estimated from the gas exchange and fluorescence methods to show the CO_2_ diffusion within the mesophyll cell level. Mesophyll conductance in flag leaves of H217, IRAT10, and H138 were 92, 88, and 58%, respectively, significantly higher than Sasanishiki (0.26 mol m^–2^ s^–1^). Hence, CO_2_ concentration within the chloroplast (Cc) in flag leaves of IRAT10 (269 μmol mol^–2^), H138 (263 μmol mol^–2^), and H217 (268 μmol mol^–2^) were remarkably higher than Sasanishiki (221 μmol mol^–2^). A significant higher electron transport rate (J) was observed in IRAT10 (190 μmol m^–2^ s^–1^), H138 (185 μmol m^–2^ s^–1^), and H217 (189 μmol m^–2^ s^–1^) flag leaves than in Sasanishiki (173 μmol m^–2^ s^–1^).

Since a higher electron transport rate was recorded in H138 and H217, genes related to photosynthesis were checked. Genes encoding light-harvesting chlorophyll a/b protein at PSII and PSI, *Lhcb1*, *Lhcb2*, *Lhcb3, Lhca1*, *Lhca2*, and *Lhca3* were significantly upregulated in H138 and H217 than in Sasanishiki and IRAT10 at the heading stage (Supplementary Figure 4). The expression level of genes encoding PSII and PSI core subunit, *psbA*, *psbD*, and *psaA*, as well as genes encoding oxygen-evolving enhancer protein 1, *psbO*, were also upregulated in H138 and H217. This facilitates a better delivery in the electronic transport chain.

**FIGURE 5:**
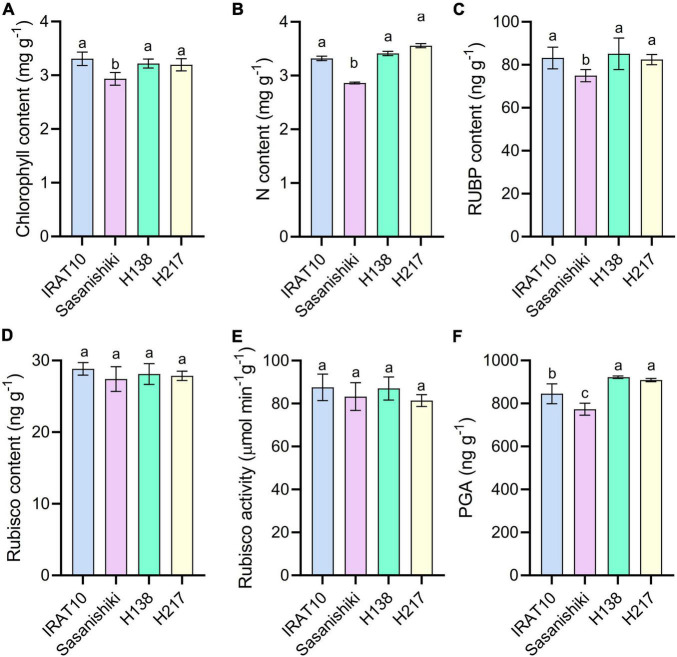
Chlorophyll content **(A)**, N content **(B)**, RuBP content **(C)**, Rubisco content **(D)**, Rubisco activity **(E)**, and PGA content **(F)** in the flag leaves of tested varieties. Values are means ± standard error. Different letters indicate significant statistical differences among tested varieties at the 0.05 probability level.

Significant higher chlorophyll content, N content, and RuBP content were observed in IRAT10 (3.3 mg g^–1^, 3.3 mg g^–1^, 83.2 ng g^–1^), H138 (3.2 mg g^–1^, 3.4 mg g^–1^, 85.1 ng g^–1^), and H217 (3.2 mg g^–1^, 3.5 mg g^–1^, 82.4 ng g^–1^) flag leaves compared to Sasanishiki (2.9 mg g^–1^, 2.9 mg g^–1^, 74.5 ng g^–1^) (Figure 5). However, Rubisco content and Rubisco activity have no difference among tested varieties. The 3-phosphoglycerate (PGA) content in IRAT10 (845 ng g^–1^), H138 (921 ng g^–1^), and H217 (908 ng g^–1^) flag leaves were significantly higher than in Sasanishiki (773 ng g^–1^).

**FIGURE 6:**
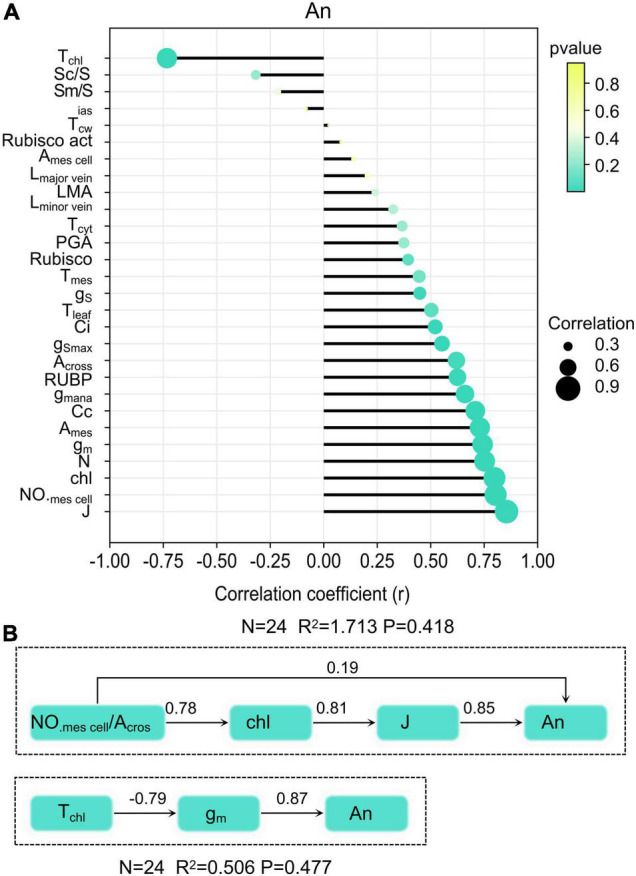
Relationship between leaf traits and An **(A)**, and the structural equation model of leaf anatomy on An **(B)**.

Anatomical traits and biochemical traits and CO_2_ diffusion traits were selected to explore the relationship between them and An. Significant higher positive correlations were observed for An with J (r = 0.85), chlorophyll content (r = 0.79), g_m_ (r = 0.74), NO._*mescell*_/A_*cros*_ (r = 0.80), A_mes_ (r = 0.72), N (r = 0.75), Cc (r = 0.70), g_m *ana*_ (r = 0.66), RuBP (r = 0.63), A_*cross*_ (r = 0.62), Ci (r = 0.52), and g_S_ (r = 0.45) (Figure 6A). Significant negative correlations were found between T_chl_ and An, cause T_chl_ was the main factor that constrains CO_2_ diffusion within mesophyll cells. Results from the structural equation model showed that mesophyll cell number has positive direct and indirect influences on An (Figure 6B). Another leaf anatomy trait that exerts an indirect influence on An was chloroplast thickness. The corresponding thickness of each component in the CO_2_ diffusion pathways has direct effects on g_m_. In tested rice, T_chl_ in the CO_2_ diffusion pathways at the liquid phase was much thicker than other components.

### DEGs Annotation and Functional Categorization

**FIGURE 7:**
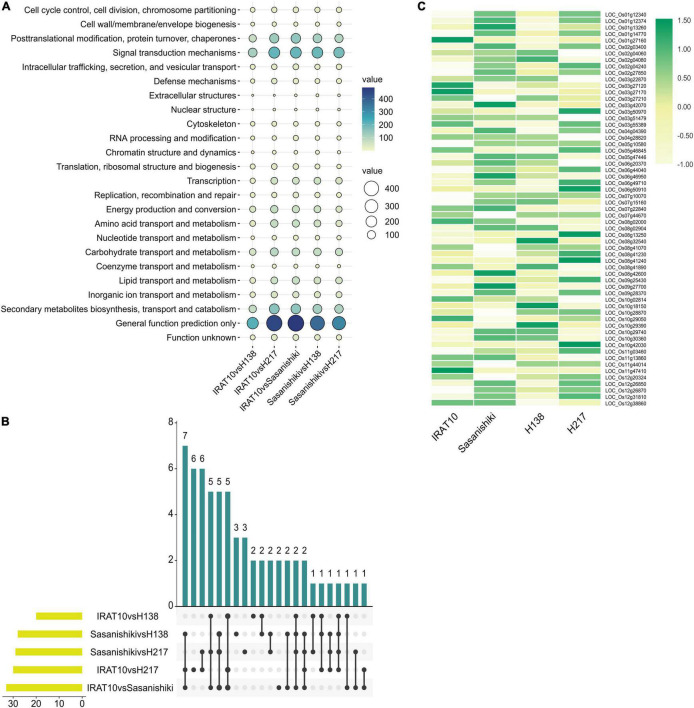
Transcriptional analysis of flag leaves among tested varieties at heading stage. KOG function classification of differentially expressed genes **(A)**. The number of differentially expressed genes from different pairwise comparisons in cell cycle classification **(B)**. Gene expression levels of genes related to cell cycle, cell division, and chromosome partitioning **(C)**.

The KOG enrichment analysis was performed on the DEGs obtained from five pairwise comparisons of transcriptomes (Figure 7). Genes related to cell cycle control, cell division, and chromosome partitioning analyzed primarily, due to differences in mesophyll cell anatomy, were observed between Sasanishiki and IRAT10, H138, and H217. A total of 61 genes in this classification were observed in the five pairwise comparisons of transcriptomes. Five common DEGs were recorded in comparisons; IRAT10 vs. Sasanishiki, Sasanishiki vs. H138, and Sasanishiki vs. H217. They are *LOC_Os02g04080* (chromosome segregation protein sudA, putative, and expressed), *LOC_Os08g41070* (retrotransposon protein, putative, unclassified, and expressed), *LOC_Os09g27700* (microtubule-associated protein, putative, and expressed), *LOC_Os11g44014* (retrotransposon protein, putative, unclassified, and expressed), *LOC_Os12g20324* (cyclin-A1, putative, and expressed) (Figure 7B). Additionally, DEGs *LOC_Os02g27850* (retrotransposon protein, putative, unclassified, and expressed), *LOC_Os05g10580* (cullin family domain-containing protein, putative, and expressed), *LOC_Os06g44040* (DOMON domain-containing protein, and expressed), *LOC_Os07g10070* (retrotransposon protein, putative, unclassified, and expressed), and *LOC_Os11g47410* (retrotransposon protein, putative, unclassified, and expressed) were recorded in comparisons IRAT10 vs. Sasanishiki, IRAT10 vs. H138, and IRAT10 vs. H217. Cyclins and cyclin-dependent kinases (CDKs) are positive regulators of cell proliferation (Fisher et al., 2012). *LOC_Os12g20324* in H138 and H217 were inherited from their parent IRAT10 and were expressed higher than their parent Sasanishiki (Figure 7C). However, *LOC_Os11g13860* (cyclin-dependent kinase, putative, and expressed) were lowly expressed in H138 and H217. The *LOC_Os08g42600* (*OsRBR1*) in H138 and H217 were inherited from their parent IRAT10 and were lowly expressed, which may contribute to small mesophyll cells and larger mesophyll cell numbers in H138 and H217 flag leaves.

## Discussion

### Higher Assimilation in Selected Rice Recombinant Inbred Lines and Related Traits

Leaves exposed to complex environmental conditions of different temperatures and different irradiations inside the canopy often show dramatically different photosynthetic properties (Huang et al., 2021). Additionally, leaf ontogeny also affects leaf photosynthetic performance (Bielczynski et al., 2017). However, higher An in flag leaves of IRAT10, H138, and H217 than in Sasanishiki is determined by genotype, because similar trends were recorded two years at the heading stage (data measured in 2019 years are shown in Supplementary Figure 5). Increasing photosynthetic rates of individual leaves within the canopy has become an important strategy for increases in crop yield potential and productivity at the condition of canopy architecture close to optimal, especially in rice (Peter, 2000; Hubbart et al., 2007). Our results provide affirmative support for this strategy, higher yields were recorded in IRAT10, H138, and H217 compared to Sasanishiki (Supplementary Table 2). Thus, understanding the underlying traits that drive higher An in selected recombinant inbred lines is crucial.

**FIGURE 8:**
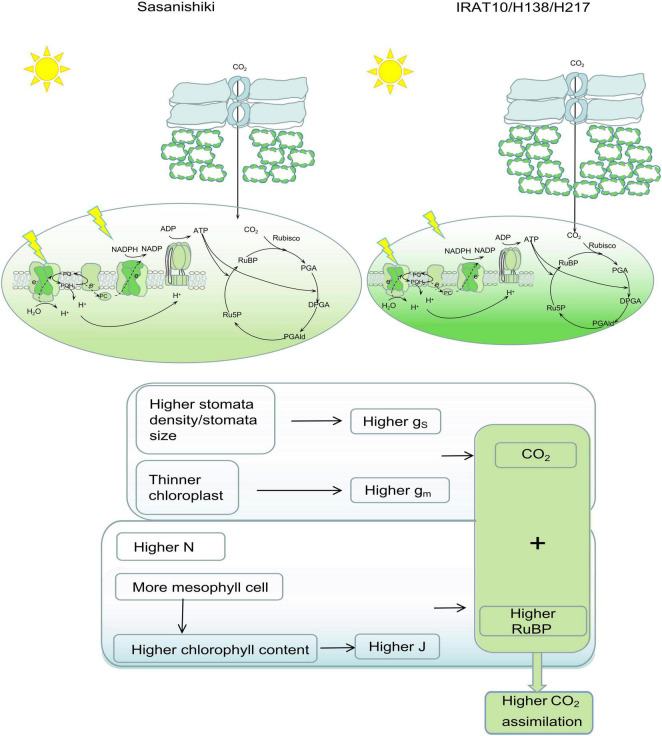
Leaf anatomy, photochemical, and biochemical traits contribute to photosynthetic differences between Sasanishiki and IRAT10, H138, and H217. Higher stomatal density/size together with thinner chloroplast contributed to higher CO_2_ concentration at the carboxylation set. More mesophyll cells, higher content, and higher chlorophyll content contributed to a higher electron transport rate, which facilitates RuBP circulation. Higher CO_2_ concentration together with higher RuBP content contributed to a higher An.

Higher stomatal conductance (g_S_) and mesophyll conductance (g_m_) in flag leaves were observed in IRAT10, H138, and H217 compared to Sasanishiki, though differences existed in achieving higher g_S_ and gm among tested varieties. This significantly facilitates higher CO_2_ concentration in intercellular spaces, as well as at the carboxylation site, higher Ci and Cc were observed in IRAT10, H138, and H217 (Figure 4). Meanwhile, higher chlorophyll content and J were recorded in IRAT10, H138, and H217 flag leaves than in Sasanishiki, which would facilitate RuBP regeneration, higher RuBP were recorded in IRAT10, H138, and H217 flag leaves (Figure 5C). Though N content in IRAT10, H138, and H217 flag leaves was higher than in Sasanishiki, no difference was observed in Rubisco content and activity among tested varieties (Figures 5A,D,E). The limitation of Rubisco activity primarily occurs at low intercellular CO_2_ concentrations (Sharkey et al., 2007; Parry et al., 2013; von Caemmerer, 2020). A higher concentration of CO_2_ and the content of RuBP at the active site of Rubisco are possibly resisting limitations from Rubisco (Parry et al., 2013; Lin et al., 2014; Sharwood, 2017). In our study, higher RuBP content together with higher CO_2_ concentration at the carboxylation site in IRAT10, H138, and H217 flag leaves promoted CO_2_ assimilation (Figure 8).

### Target to Modify Leaf Anatomy for Achieving Higher Assimilation in Rice

Leaf anatomy, exerting influence on light use efficiency and CO_2_ diffusion, is considered to have dramatic potential for photosynthesis performance increase. Also, increase cell density in the mesophyll of Arabidopsis leaf does increase leaf photosynthetic capacity (Lehmeier et al., 2017). Our result showed that NO._*mescell*_/A_*cros*_ exert positive influences on An through chlorophyll content (Figure 6B). Higher chlorophyll content and larger antenna size could intercept and absorb more light energy (Ort et al., 2015). In this study, IRAT10, H138, and H217 have higher mesophyll cell numbers when compared to Sasanishiki flag leaves. Simultaneously, higher chlorophyll content and expression of genes encoding the light-harvesting protein of PSII and PSI (*Lhcb1*, *2*, *3* and *Lhca1*, *2*, *3*) were recorded in IRAT10, H138, and H217, which facilitates light use efficiency. Better light use efficiency may facilitate RuBP regeneration and promotes CO_2_ assimilation. Fortunately, cell density can be manipulated by modifying cell cycle-associated genes. Retinoblastoma-related gene, *RBR1*, plays a central role in regulating gene expression, endoreduplication, the number, size, and death (Sabelli et al., 2013). In Arabidopsis, suppression of *RBR1* can be used to generate organs with smaller cells in leaves (Lehmeier et al., 2017). Gene *OsRBR1* (*LOC_OS08g42600*) in H138 and H217 were inherited from IRAT10 and were lowly expressed than IRAT10 (Figure 7B). This might cause a larger number of small mesophyll cells in H138 and H217 flag leaves. Hence, mesophyll cell density will be a good target to improve photosynthesis in engineering leaf anatomy.

### Relationship of gm to Leaf Anatomy Traits in Rice

Harley’s methods and anatomical methods were used to esstimate g_m_ as accurately as possible. A significantly higher positive correlation was found between the two methods, suggesting that somehow the variations in gm among the tested varieties are at least partly related to the differences in the thickness of the structures involved in CO_2_ diffusion pathways (Tomas et al., 2013). Despite the same variation of g_m_ in tested breeds being observed, a significantly lower value was recorded in anatomy methods compared to Variable J methods. This indicates that the resistance derived from anatomy structure is limited or is underestimated, more properties related to each component need to be considered in modeling gm. For one reason, the distance from the bottom of the bulfom cell to the outermost mesophyll cell is about half the distance between the two epidermis cells due to the presence of bulfom cells. In this paper, the averaged distance of the two distances mentioned above was taken as the mesophyll layer thickness. Additionally, g_m_ cannot be regarded as a pure diffusion component, gm is essentially a flux-weighted quantity, the amount of CO_2_ diffused from mitochondria to chloroplasts at the process of respiration and photorespiration had to be considered in gm estimation (Tholen et al., 2012).

Considering only diffusional component, limitation derived from the liquid phase components was the main factor constraining CO_2_ diffusion in tested varieties, which is consistent with the results found in cotton and gymnosperms (Han et al., 2018; Carriqui et al., 2020). Interestingly, T_chl_ was the main factor restricting CO_2_ diffusion in liquid-phase in tested rice, rather than T_cw_. The corresponding thickness of each component in the CO_2_ diffusion pathways has direct effects on mesophyll conductance. In tested rice, the chloroplast thickness was significantly thicker than the cell wall thickness of mesophyll cells. Cell wall resistance to CO_2_ diffusion depends on physical properties like cell wall thickness, porosity, and tortuosity. Meanwhile, cell wall porosity varied with T_cw_, and thinner cell wall at mesophyll cells may with a higher cell wall porosity Tosens et al., 2016. Cell wall thickness in tested varieties was thinner than that reported in cotton and gymnosperms (Han et al., 2018; Carriqui et al., 2020). Additionally, cell wall thickness, conformation, and complexity can be modified by changing cell wall components and biochemical properties, which exert dramatic influences on CO_2_ diffusion through the cell wall (Carriqui et al., 2020). Hence, T_cw_ may play only a minor role in constraining CO_2_ diffusion in rice flag leaves. Chloroplast thickness was the dominant factor restricting CO_2_ diffusion within mesophyll cells rather than cell wall thickness.

Previously, LMA was reported as negatively correlated with gm, but this relationship has been redefined (Ren et al., 2019). They reviewed that an increase in LMA dramatically improves Sm and Sc when LMA is less than 100 g m^–2^, but plays only a minor role when it exceeds 100 g m^–2^, even higher LMA reduces the *f*_*ias*_, Sc/Sm, and gm values (Ren et al., 2019). In our study, LMA was far less than 100 g m^–2^ in all tested varieties, with IRAT10 having the highest and H217 having the lowest, but we did not observe an increase in Sm and Sc consistent with LMA (Tables 1, 2). On the contrary, Sm and Sc in IRAT10 flag leaves were significantly lower than that of Sasanishiki, H138, and H217. This is mainly due to IRAT10 having a larger mesophyll cell size and more mesophyll cell numbers, but lower intercellular airspace within leaves (Tables 1, 2). The result indicated that rice, different from cotton and gymnosperms, is a special species. An increase in LMA may lead to the dense packing of mesophyll cells, which dramatically reduces Sm/S and inevitably decreases gm (Flexas et al., 2008; Niinemets et al., 2009; Sarathi et al., 2016). Reasonably, we observed a significant negative correlation of Sm and Sc with g_m_. Also, the slice photos were shown in Supplementary Figure 6. Hence, the difference among tested varieties can be mainly explained by the difference observed in chloroplast thickness.

In summary, we analyzed the photosynthetic capacity and corresponding constraints on CO_2_ assimilation in selected rice recombinant inbred lines, H138 and H217, compared to their parent. Higher chlorophyll content and electron transport rate (mediated by higher mesophyll cell numbers), together with higher mesophyll conductance (mediated by chloroplast thickness), contributed to higher An in flag leaves of H138 and H217 at the heading stage. Chloroplast thickness was the dominant factor restricting CO_2_ diffusion within mesophyll cells rather than cell wall thickness. Mesophyll cell density will be a good target to improve photosynthesis in engineering leaf anatomy.

## Data Availability Statement

The datasets presented in this study can be found in online repositories. The names of the repository/repositories and accession number(s) can be found below: NCBI; PRJNA834750.

## Author Contributions

DM and JW designed experiments. FZ, LZ, and YL performed experiments. FZ, LZ, and GW analyzed data and compiled figures. FZ and JW wrote the manuscript. DM edited the final manuscript. All authors contributed to the article and approved the submitted version.

## Conflict of Interest

The authors declare that the research was conducted in the absence of any commercial or financial relationships that could be construed as a potential conflict of interest.

## Publisher’s Note

All claims expressed in this article are solely those of the authors and do not necessarily represent those of their affiliated organizations, or those of the publisher, the editors and the reviewers. Any product that may be evaluated in this article, or claim that may be made by its manufacturer, is not guaranteed or endorsed by the publisher.

## Footnotes

1
https://rapdb.dna.affrc.go.jp/index.html
