# Supplementary material for: Higher CO2 Assimilation in Selected Rice Recombinant Inbred Lines Is Driven by Higher CO2 Diffusion and Light Use Efficiency Related to Leaf Anatomy and Mesophyll Cell Density
Source: Front Plant Sci. 2022 Jun 9;13:915050. doi: 10.3389/fpls.2022.915050 (PMC9261980; doi:10.3389/fpls.2022.915050)

Supplementary Material

# Supplementary Tables

**Supplementary Table 1.** Primers used in this study for qPCR

|  | Primer | Primer Sequence | Gene description |
| --- | --- | --- | --- |
| *Lhcb1* | Forward | GCAGTAGATGTACGTACGTGTA | Chlorophyll A-B binding protein |
|  | Reverse | ACCTGCACAGAAATACACACTA |  |
| *Lhcb3* | Forward | TGTGTACACTTGTAGTAGCCAC | Chlorophyll A-B binding protein |
|  | Reverse | CACACACACACACAATTAAGGT |  |
| *Lhcb2* | Forward | GATGGCGTCCACGATCAT | Chlorophyll A-B binding protein |
|  | Reverse | GTACTTCACCCTATCCGGC |  |
| *Lhca1* | Forward | CATCATCCCGAGAACCATCTAC | Chlorophyll A-B binding protein |
|  | Reverse | CACATAAAAACCACCCATAGCC |  |
| *Lhca2* | Forward | AAACGTAATGTTCAGGTACCCT | Chlorophyll A-B binding protein |
|  | Reverse | TAGTGTTTATGGACTACGGACC |  |
| *Lhca3* | Forward | CTACGGCGAGGTGTTCAA | Chlorophyll A-B binding protein |
|  | Reverse | TCGAAGACGAAGAGAGTGTAAG |  |
| *psbA* | Forward | CCCTCATTAGCAGATTCGTTTT | Photosystem II reaction center protein A |
|  | Reverse | ATGATTGTATTCCAGGCAGAGC |  |
| *psbD* | Forward | AACCGCAGCAGTTTCCACC | Photosystem II reaction center protein D |
|  | Reverse | GCACCATCACCGTCCTCAA |  |
| *psbO* | Forward | GAACCATCAAGTTCGAGGAGAA | Oxygen-evolving enhancer protein 1 |
|  | Reverse | ACCAGATTCTTGATGGTGAAGA |  |
| *psaA* | Forward | GCGAGCAAATAAAACACCTTTC | Photosystem I core proteins A |
|  | Reverse | GTACCAGCTTAACGTGGGGAG |  |
| *Ubiqitin* | Forward | GCTCCGTGGCGGTATCAT | Reference gene |
|  | Reverse | CGGCAGTTGACAGCCCTAG |  |

**Supplementary Table 2.** Yield and yield components of tested varieties. Values are means ± standard error. Different letters indicate significant statistical differences among tested varieties at the 0.05 probability level.

| Genotype | IRAT10 | Sasanishiki | H138 | H217 |
| --- | --- | --- | --- | --- |
| Panicle number per hill | 16.4±1.5 b | 24.3±1.5 a | 25.5±2.5 a | 24.7±2.1 a |
| Spikelets per panicle | 186.4±32.6 a | 110.8±7.2 c | 138.9±22.1 b | 143.3±31.8 b |
| Grain-filling ratio (%) | 82.2±3.7 b | 87.2±2.1 a | 81.9±3.4 b | 80.5±6.7 b |
| 1000-grain weight (g) | 24.7±1.7 b | 24.1±0.9 b | 26.6±1.5 a | 25.2±1.6 ab |
| Grain weight per hill | 52.8±3.7 a | 41.8±2.1 c | 45.8±2.5 b | 47.1±4.6 b |

# Supplementary Figures

**Supplementary Figure 1.** The genetic map of the two recombinant inbred lines. AA

**
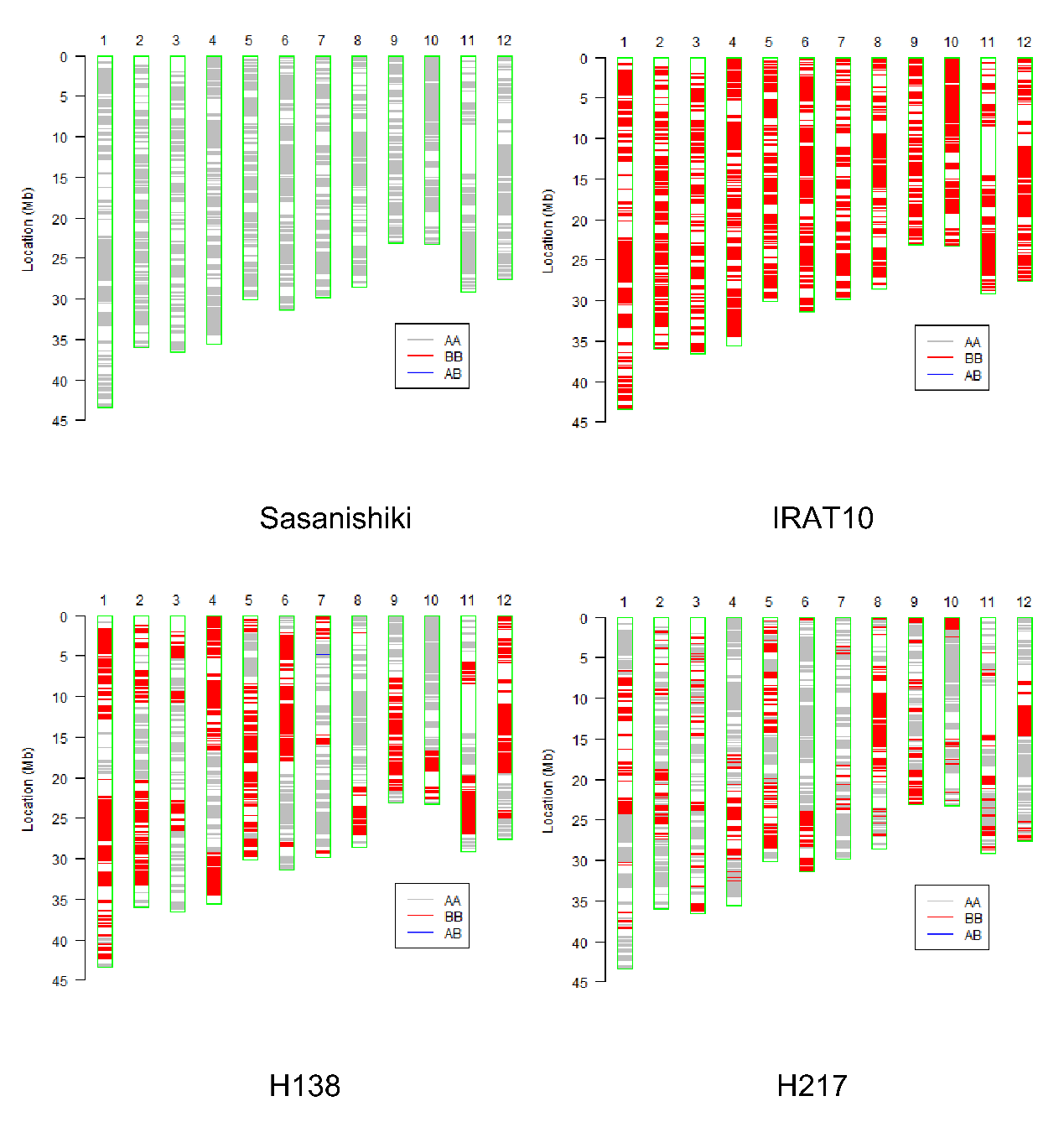
**

**Supplementary Figure 2.** Transmission electron microscopic images of the chloroplast ultrastructure. Cp, chloroplast; Thy, thylakoid lamellae; OB, osmophilic body; SG, starch granule.


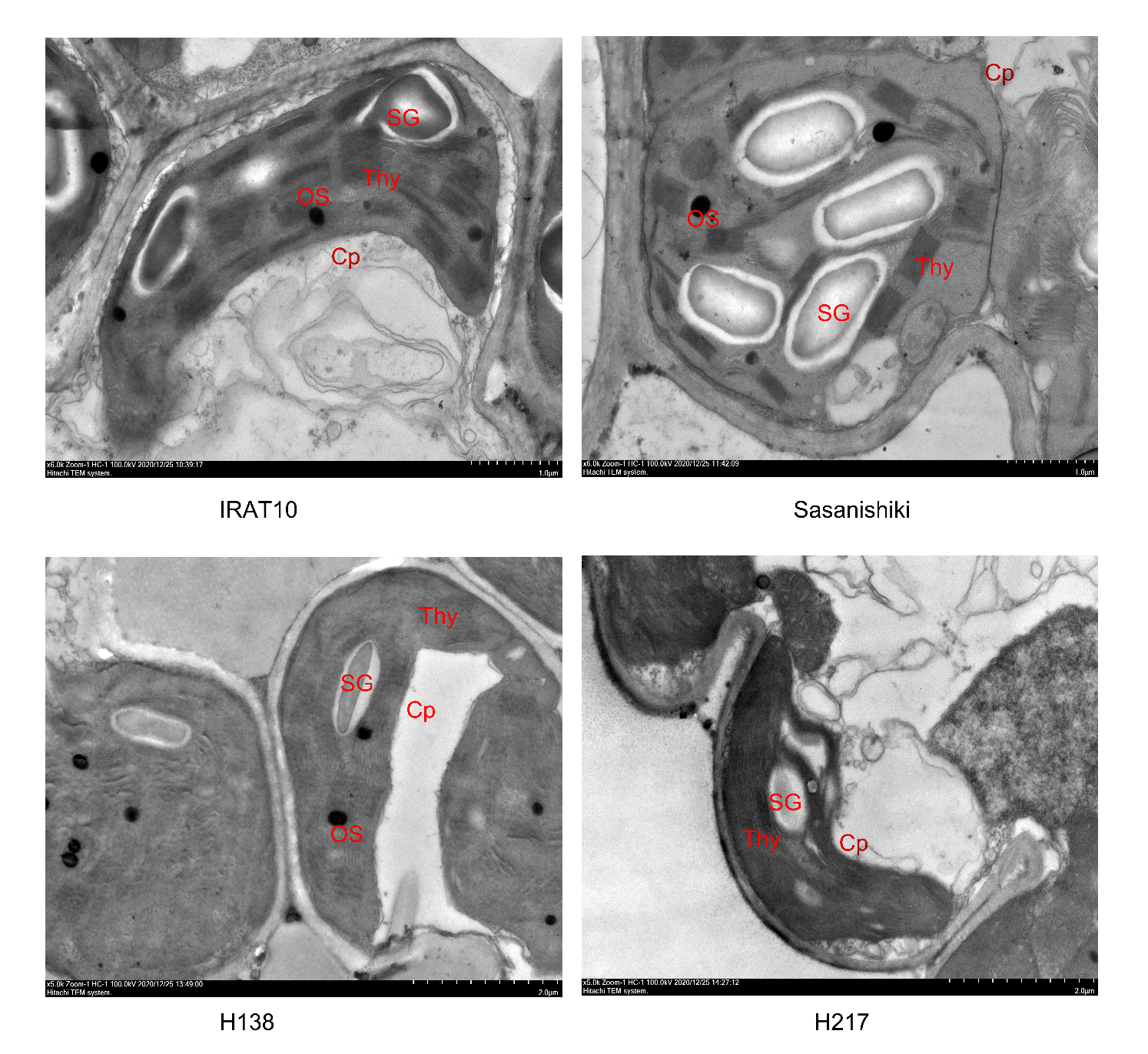


**Supplementary Figure 3.** Stomatal pores on both abaxial and adaxial sides, only IRAT10 (left) and Sasanishiki were shown. Red squares indicate stomata pores.


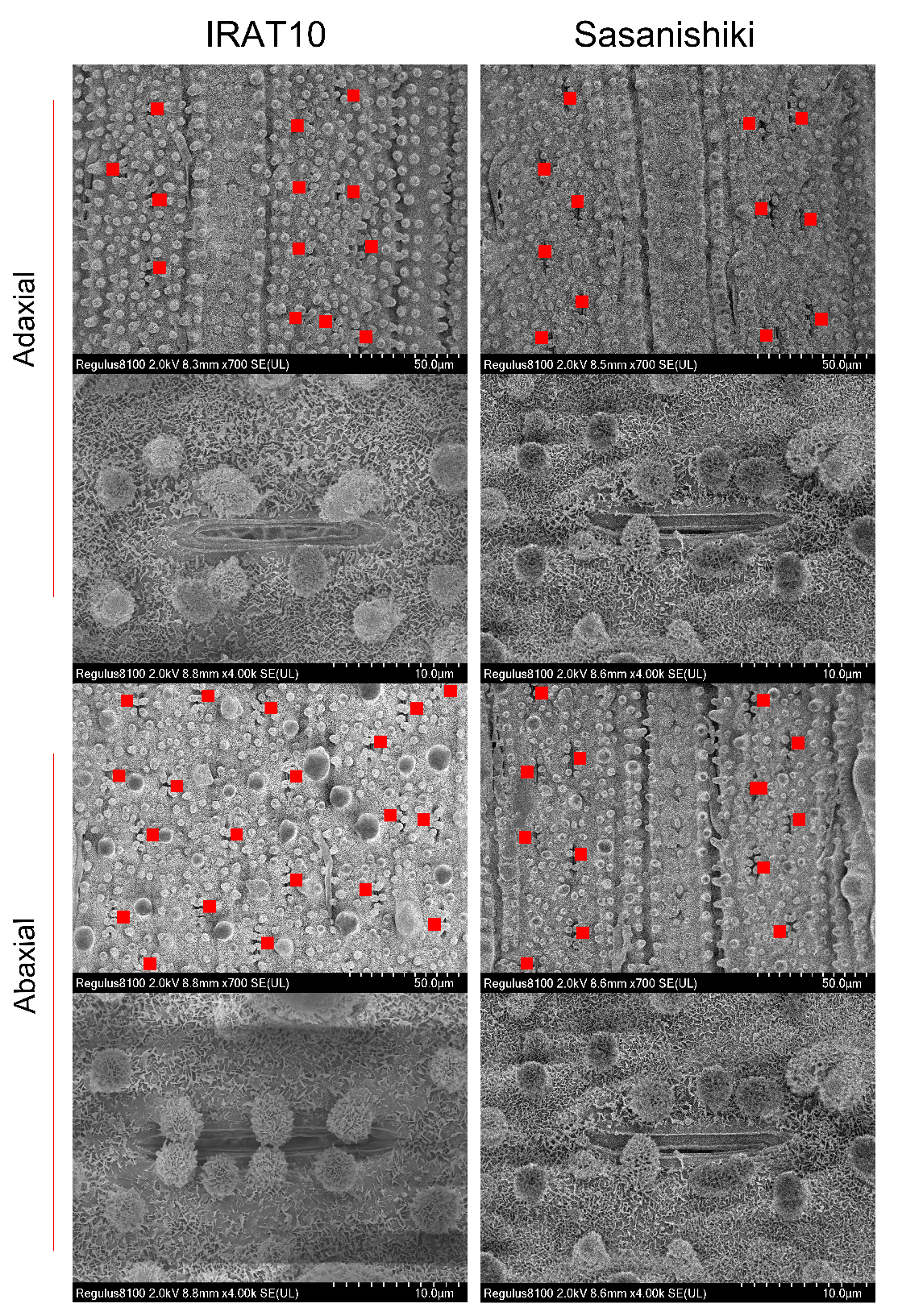


**Supplementary Figure 4.** Expression parent of genes related to photosynthesis of tested varieties at heading stage. Values are means ± standard error. Different letters indicate significant statistical differences among tested varieties at the 0.05 probability level.


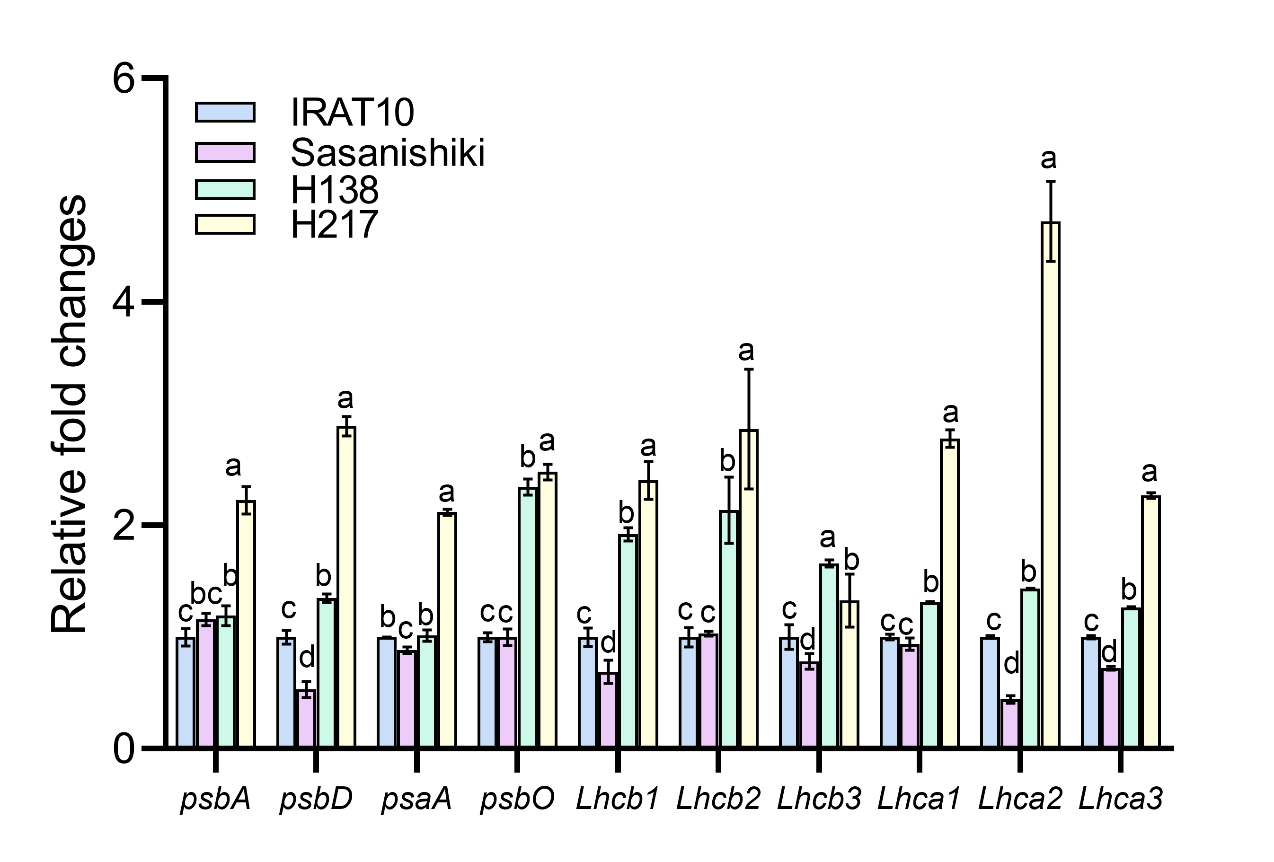


**Supplementary Figure 5.** Gas exchange measurement. Net CO_2_ assimilation rate (An) (*A*), stomatal conductance to water (gs) (*B*) intercellular CO_2_ concentration (Ci) (*C*) mesophyll conductance (gm) (*D*), and CO_2_ concentration within the chloroplast envelope (Cc) (*E*). Data were measured in 2019. Values are means ± standard error (n=6). Different letters indicate significant statistical differences among tested varieties at the 0.05 probability level.


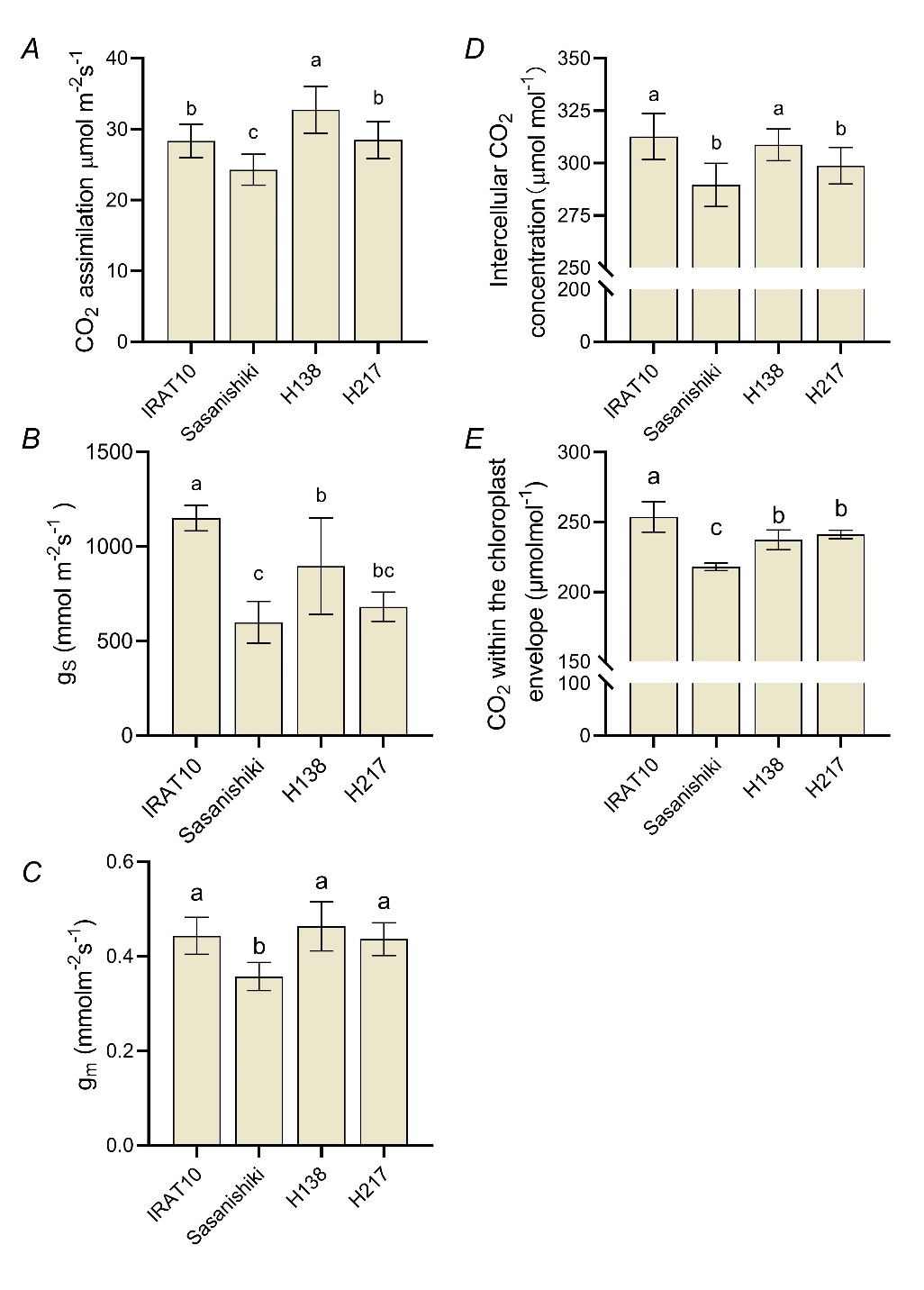


**Supplementary Figure 6.** Longitudinal slice images of flag leaves of tested rice, only IRAT10 (right) and Sasanishiki (left) were shown.


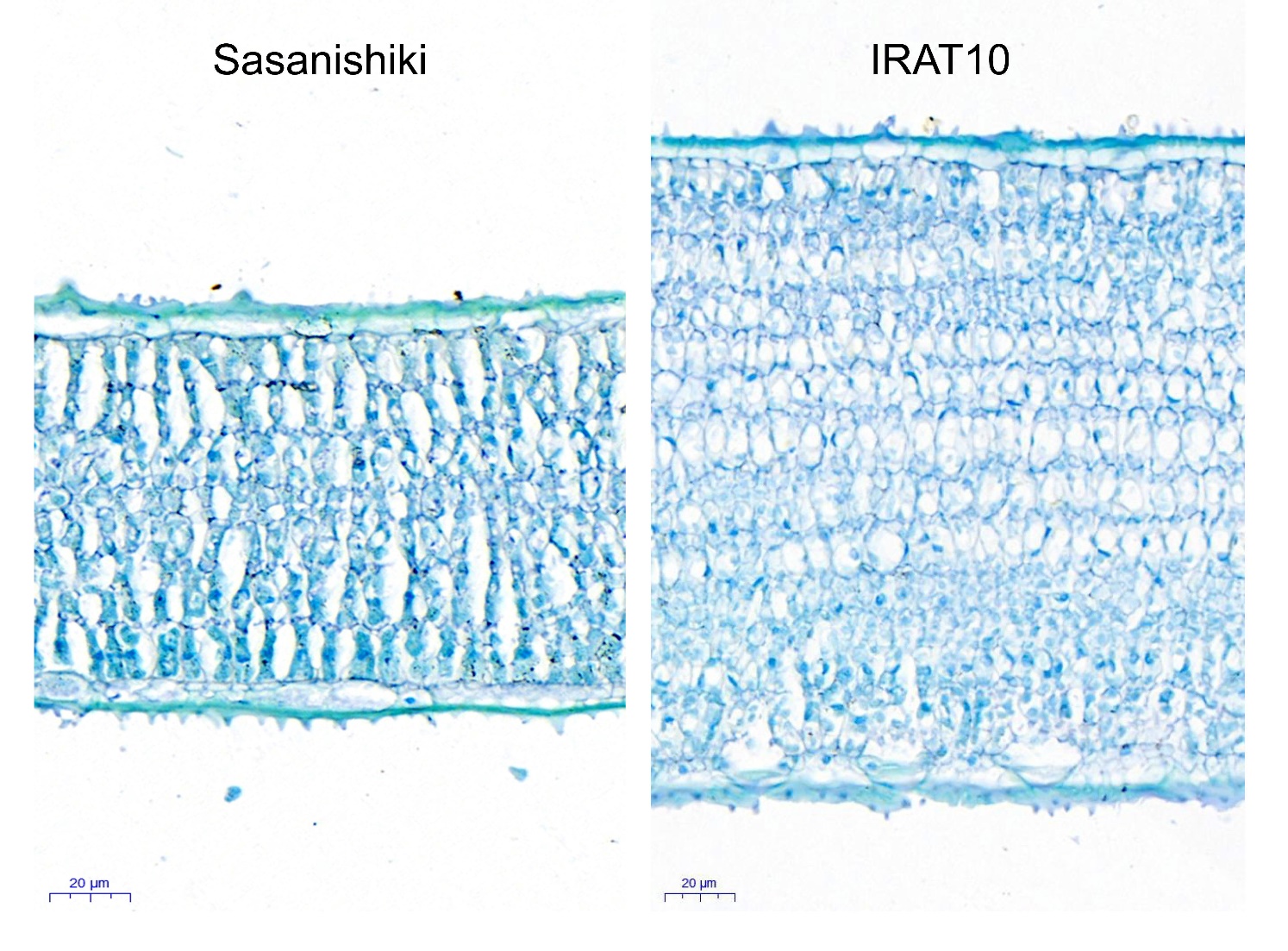

Supplement: Supplementary file 1 [file Data_Sheet_1.docx]
